# Dynamics of Bacterial Communities by Apple Tissue: Implications for Apple Health

**DOI:** 10.4014/jmb.2305.05003

**Published:** 2023-07-07

**Authors:** Hwa-Jung Lee, Su-Hyeon Kim, Da-Ran Kim, Gyeongjun Cho, Youn-Sig Kwak

**Affiliations:** 1 Division of Applied Life Science (BK21Plus), Gyeongsang National University, Jinju 52828, Republic of Korea; 2 Department of Plant Medicine, Research Institute of Life Science, Gyeongsang National University, Jinju 52828, Republic of Korea; 3 Division of Agricultural Microbiology, National Institute of Agriculture Science, Rural Development Administration, Wanju 55365, Republic of Korea

**Keywords:** Biological control, *Erwinia amylovora*, keystone taxa, *Malus pumila*, microbial community

## Abstract

Herein, we explored the potential of the apple's core microbiota for biological control of *Erwinia amylovora*, which causes fire blight disease, and analyzed the structure of the apple's bacterial community across different tissues and seasons. Network analysis results showed distinct differences in bacterial community composition between the endosphere and rhizosphere of healthy apples, and eight taxa were identified as negatively correlated with *E. amylovora*, indicating their potential key role in a new control strategy against the pathogen. This study highlights the critical role of the apple's bacterial community in disease control and provides a new direction for future research in apple production. In addition, the findings suggest that using the composition of the apple's core taxa as a biological control strategy could be an effective alternative to traditional chemical control methods, which have been proven futile and environmentally harmful.

## Introduction

The apple (*Malus pumila* M.) is an economically significant fruit crop, the popularity of which is attributed to the fruit’s desirable flavor, sugar-acid content, metabolites, aroma, and overall texture and palatability. Additionally, apples are a rich source of nutrients, including antioxidants, vitamins, and dietary fiber [1]. As a result, apples are widely enjoyed, with 83 million tons consumed globally. In 2020, China was the largest producer of apples with 40.5 million tons, followed by the United States (4.6 million tons), Turkey (4.3 million tons), India (2.3 million tons), and Russia (1.5 million tons) [2].

A range of diseases affecting apples, including fire blight, anthracnose, marssonia blotch, powdery mildew, scab, crown rot, Rosellinia root rot, and replant disease have been commonly reported in the literature [3,4]. Fire blight, caused by *Erwinia amylovora*, is a highly destructive disease that affects rosaceous plants, such as apples, pears, and raspberries [5]. This disease leads to significant annual economic losses by reducing production and quality [6]. The symptoms of fire blight include discoloration ranging from reddish to black on flowers, fruits, leaves, and twigs. If left unchecked, the entire plant may succumb to the disease in severe cases. Infected branches, fruits, and twigs may also exhibit a watery ooze during periods of high humidity [7]. During the flowering stage of infection by *E. amylovora*, secondary infections may occur in wounds and flowers of the plant via insect vectors, wind, or rain [8, 9]. Fire blight disease is being controlled in many countries through the implementation of phytosanitary measures, such as the disposal of all infected trees by burying them to prevent the disease from spreading [10]. Antibiotics and chemical pesticides, such as streptomycin and oxytetracycline, are primarily utilized for the control of fire blight disease. However, there has been a recent emergence of strains resistant to these antibiotics and pesticides and there are also reports of phytotoxicity [10, 11]. Thus, biological control methods through microbiota research are gaining attention as an alternative solution.

Apple trees host a consortium of microbial communities, including bacteria, fungi, protists, viruses, and nematodes, that colonize the surface and internal tissues of the plant [12]. The coevolution of these microbiota communities with their host plants results in the formation of a plant microbiome [13, 14]. Over the last few decades, numerous studies have recognized the key role of microorganisms in natural ecosystems [15-18]. The plant microbiome significantly impacts plant health and productivity, including nutrient acquisition, abiotic stress tolerance, biotic stress resilience, and host immune regulation [19, 20]. The plant microbiota can colonize various regions of the plant, including the rhizosphere, endosphere, and episphere. The rhizosphere, which is a narrow region of soil influenced by root exudates, exhibits the highest abundance and diversity of microbiota [21]. Microorganisms that colonize the interior of the plant, referred to as the endosphere, can have a direct impact on the evolution of the plant. Additionally, the microbial community outside of plant tissues, the episphere, can also elicit plant immune responses and provide protection against pathogens [22]. Therefore, the microbiome plays a crucial role in maintaining the health of the plant in various regions.

The advancement of next-generation sequencing technologies has provided new insights into the microbial ecology of several crops, including grapes [23, 24], olive [25], mango [26], and apple [27]. Despite the importance of understanding microbial diversity in crops, studies on the microbial diversity of apples according to tissue and developmental stage have yet to be conducted. To address this gap in knowledge, we conducted a comprehensive analysis of the apple microbiota communities based on tissue organization and growth stage. Our results will contribute to the field of microbiome research in *M. domestica* and provide fundamental knowledge for the development of Synthetic Communities (SynCom) to prevent and control the fire blight pathogen, *E. amylovora*.

## Materials and Methods

### Sampling and Experimental Design

An orchard of apples (cv. Fuji, 20-year-old) located in Eumseong, Korea (36°56'46.3"N 127°43'13.0"E) was sampled a total of five times (Table S1). Rhizosphere soils were collected from sampling 1 to sampling 5, while endosphere (leaf bud, flower bud, flower, twig, fruit) and episphere (leaf bud, flower bud, flower, leaf, fruit) samples were collected according to the stages of apple development (Fig. S1). To investigate tissue- and seasonal microbial community structures, rhizosphere, episphere, and endosphere samples were collected from five selected trees at each growth stage (Fig. S2). Rhizosphere soil samples (*n* = 5) were collected from the root surface in 1-3 mm. Tissue samples (leaf buds, flower buds, flowers, twigs, leaves, and fruits) were taken from each tree (leaf buds: 1.9-2.5 g, flower buds: 2-3 g, flowers: 2-4.5 g, twigs: 1.5 g, leaves: 5 g, and fruits: 1.5 g) and immediately placed on ice to preserve microbial communities. Finally, bee gut samples (*n* = 3) were collected from bee-pollinated flowers during the developing flower stage (2nd and 3rd samplings).

### Microbial DNA Extraction

For DNA extraction, in both the episphere and endosphere, tissues were subjected to a 1 × PBS buffer (prepared from 10 × PBS solution containing 80 g of NaCl, 2 g of KCl, 1.44 g of Na2HPO_4_, 2.4 g of KH_2_PO_4_, adjusted to a pH of 7.4 with a final volume of 1 L) in 50 ml Falcon tubes. The samples were sonicated (Elmasonic S150, USA) at 35 kHz for 45 s, followed by vortexing for 30 s, repeated three times. After sonication, the supernatant, which included leaf bud, flower bud, flower, leaf, and fruit, was transferred to a 50 ml Falcon tube and utilized for the preparation of episphere samples. Prior to DNA extraction, the episphere samples were centrifuged at 2,000 ×*g* and 4°C for 20 min, and the supernatant was discarded with the exception of 10 ml. The endosphere samples were subjected to sterilization by immersion in 70% ethanol for 30 s, 1% NaOCl for 30 s, and ddH_2_O for 2 times, before being dried for 1 h at room temperature on a clean bench. The samples were then completely dried and ground with liquid nitrogen.

The total microbial DNA was extracted according to the provided manual using the FastDNA SPIN Kit for Soil (MP Biomedicals, USA) with 0.5 g of the rhizosphere soil, 0.3 g of endosphere (buds, flower, twig, leaf, and fruit), and 0.5 ml of episphere (buds, flower, twig, leaf, and fruit). To solubilize membrane proteins, the sample was homogenized with 978 μl of sodium phosphate buffer and 122 μl of MT buffer in the FastPrep instrument at a speed of 6.0 for 40 s. After centrifugation at 14,000 ×*g* for 10 min using the Microfuge centrifuge (Beckman Coulter, Germany), the supernatant was transferred to a clean microcentrifuge tube and 250 μl of protein precipitation solution was added and inverted 10 times. The tube was centrifuged for 5 min. The supernatant was transferred to a 15 ml conical tube containing 1 ml of binding matrix, and the tube was inverted for 2 min. The conical tube was incubated for 3 min to facilitate DNA binding, and the supernatant of 500 μl was discarded. The resuspension of the supernatant and binding matrix was then transferred to an empty catch tube and centrifuged at 14,000 ×*g* for 1 min. The same process was repeated on the remaining mixture. Finally, DNA was purified and stored at -20°C, after the DNA bind matrix was washed by 50 μl of the SEWS-M.

### Library Construction for Amplicon Sequencing

For the amplification of the V3-V4 regions of the 16S rRNA gene, the DNA was diluted to a concentration of 5-8 ng/μl. The amplification was carried out using the 341F-805R primer set that included overhang adapter sequences for Miseq (TCGTCGGCAGCGTCAGATGTGTATAAGAGACAGCCTACGGGNGGCWGCAG, and GTCTCGTGGGCTCGGAGATGTGTATAAGAGACAGGACTACHVGGGTATCTAATCC) and PNA probes (pPNA and mPNA) [28]. The PNA probes were employed to eliminate potential contamination from plant-derived mitochondrial and chloroplast DNA in both endosphere and episphere samples (Table S2). The rhizosphere sample PCR was conducted with a reaction mixture consisting of 2.5 μl of genomic DNA, 1 μl of each primer (10 pmol), 20 μl of 2 × PCR buffer for KOD FX Neo, 4 μl of dNTPs (2 mM), 0.3 μl of KOD FX Neo (1.0 U/μl; Toyobo, Japan), and ddH_2_O to a final volume of 40 μl. The reaction was run on a T100™ thermal cycler (ThermoFisher, USA) using the following conditions: 95°C for 3 min (1 cycle), 95°C for 30 s, 55°C for 30 s, 72°C for 30 s (25 cycles). For endosphere and episphere samples, the reaction mixture consisted of 2.5 μl of total DNA, 1 μl of each primer (10 pmol), 2.5 μl of each PNA probe (7.5 μM), and 12.5 μl of KAPA HiFi HotStart ReadyMix (Roche, Switzerland) in a final volume of 22 μl. The reaction conditions were as follows: 95°C for 3 min (1 cycle), 95°C for 30 s, 78°C for 10 s, 55°C for 30 s, and 72°C for 30 s (25 cycles). Subsequently, the samples were purified utilizing AMPure XP (Beckman Coulter, USA) in accordance with the manufacturer's protocol. A 20 μl aliquot of PCR products was added to AMPure XP and incubated for 5 min to allow binding. The mixture was then subjected to magnetic separation, using a magnet rack, to separate the DNA fragments and beads from contaminants. The contaminants were washed twice with 200 μl of 70% ethanol. The purified DNA was eluted by adding 20 μl of 10 mM Tris-HCl (pH 8.5; Biosolution, Korea). The NGS Illumina Miseq (Illumina, USA) with MiSeq Reagent Kit v2 (500 cycles) was performed by Macrogen (Korea) and the data were acquired in the form of fastq files.

### Bacterial Community Analyses

The raw sequence was generated for the purpose of assigning Amplicon Sequence Variants (ASVs) using the DADA2 version 1.8 pipeline tutorial [29] within the R software version 4.2.1. Each read was trimmed to a length of 240 and 280 nucleotides, with a quality score higher than 30, in order to carry out the filtering process. The forward and reverse reads were merged, and chimeras were removed. Taxonomic assignment was performed using Silva version 138 (https://www.arb-silva.de/download/arb-files/) and IDTAXA [30]. Rarefaction curve analysis, alpha diversity (Observed ASV, Shannon, and Simpson), and beta diversity analysis were conducted using the ggiNEXT version 2.0.20 and phyloseq version 3.12 packages. Principal Coordinates Analysis (PCoA) was employed to visualize the similarities and dissimilarities based on the Bray-Curtis distance. In the co-occurrence network analysis, a correlation was confirmed using the SparCC (Sparse correlations for compositional data) method on genus level summarized ASVs using FastSpar version 1.0.0 (https://github.com/scwatts/fastspar). The graph visualization was performed using the ggplot2 version 3.3.3.

## Results

### Bacterial Diversity in Different Tissues of Apple

**Fig. 1:**
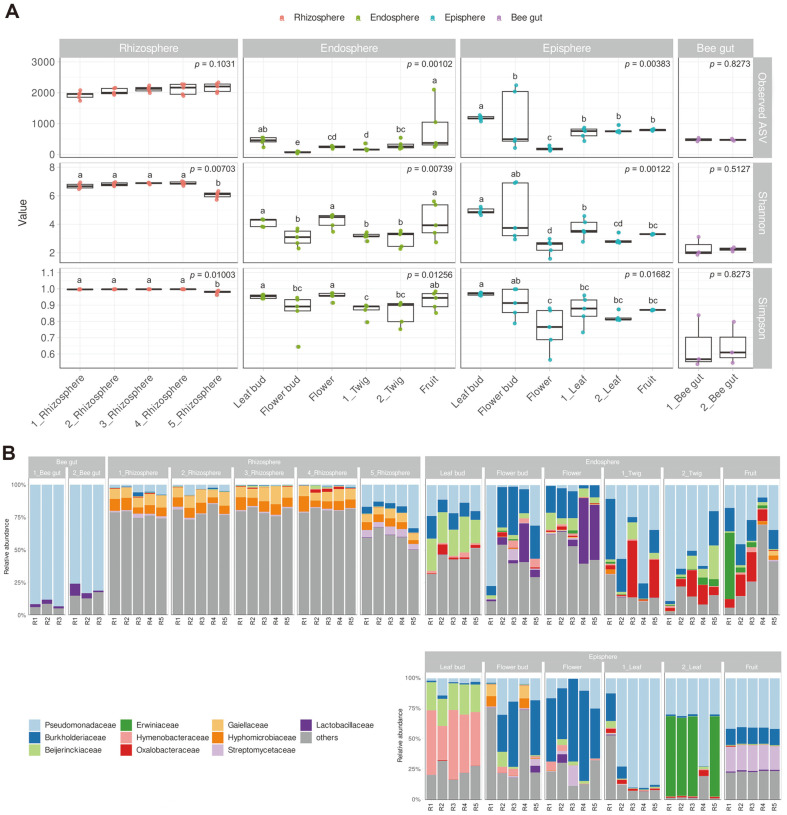
Species diversity according to tissues and growth stage. The amplicon sequence variant (ASV) was assigned by IDTAXA. (**A**) Variables (Observed ASV, Shannon, Simpson) were used for alpha diversity. Statistical significance appeared using Kruskal–Wallis test. The post-hoc analysis was conducted using the Conover-Iman test. The lowercase letters above each box whisker indicate groups with no difference by the post-hoc (*p* > 0.05). The order on the x-axis from left to right corresponds to the sampling order, and the 3 to 5 dots displayed above it correspond to each value of the replicated samples. (**B**) Relative abundance of microbial communities at the family level. The x-axis represents replication numbers for each tissue. The family level was cut off in the top 10.

The bacterial communities in apples were revealed by sequencing the V3-V4 region of 16S rRNA based on apple growth stages and tissues. A total of 4,973,610 sequence reads, which were merged by forward and reverse read complementarity, were generated (Table S3) and the taxonomic classification of microbiota sequences was done using IDTAXA to assign ASVs. GenBank accession numbers for all samples were presented in Table S4. The alpha diversity analysis showed consistent microbial numbers in the apple rhizosphere across sampling 1 to sampling 5 (Fig. 1A). However, endosphere and episphere bacterial diversities were affected by both tissues and season. Notably, the lowest diversity of the bacterial community was observed in bee gut samples (Fig. 1A). Observed ASV meaning alpha diversity of endosphere were 232-546 in leaf bud, 33-101 in flower bud, 183-280 in flower, 141-357 in twig and 246-2100 in fruit. Observed ASV of episphere values were 1070-1157 in leaf bud, 210-2242 in flower bud, 119-288 in flower, 433-953 in leaf, and 764-823 in fruit. The rhizosphere had an evenness value of 7 in the Shannon and Simpson indices, while the endosphere and episphere showed evenness values of 2-6 (Shannon) and 0.5-0.99 (Simpson) respectively, according to tissues. The relative abundance of bacteria abundance was determined based on the top 10 taxa at the family level in each tissue and season (Fig. 1B). The results showed that all tissues contained *Pseudomonadaceae*, *Burkholderiaceae*, *Beijerinckiaeceae*, *Erwiniaceae*, *Hymenobacteraceae*, *Oxalobacteraceae*, *Gaiellaceae*, *Hyphomicrobiaceae*, *Streptomycetaceae*, and *Lactobacillaceae*. In the bee gut, *Pseudomonadaceae* dominated with a relative abundance of 83-93%. In the rhizosphere (Table S5), *Gaiellaceae* and *Hyphomicrobiaceae* had relative abundances of 5-14% and 2-9%, respectively. In the top 10 genera of relative abundance in the rhizosphere, *Hyphomicrobium* and *Gaiella*, belonging to *Hyphomicrobiaceae* and *Gaiellaceae*, respectively, were identified. In the rhizosphere sampling 5, the relative abundance of *Pseudomonadaceae* was 12-34% higher compared to samplings 1 to 4. There was no significant difference observed in the bacterial composition and relative abundance between the rhizosphere and the endosphere. However, a noticeable difference was observed between the endosphere and episphere when compared to the rhizosphere. In the endosphere leaf bud, *Beijerinckiaeceae* showed the highest relative abundance, with a range of 17-28%. *Burkholderiaceae* (2-38%) was also present in high abundance in the flower bud and flower of the endosphere, compared to other tissues. On the other hand, *Ralstonia* showed the highest relative abundance in the flower bud, flower, and fruit (Table S6). As the flower developed into fruit, *Pseudomonadaceae* abundance showed to a range of 9-46%. Additionally, twigs showed a high abundance of *Pseudomonadaceae* (10-90%). Similarly, *Burkholderiaceae* (0.7-75%) existed in high abundance in the flower bud and flower of the episphere. In the leaf of the episphere, *Erwiniaceae* showed relative abundance at a range of 0.04-66% in the fourth sampling. In the top 10 relative abundance of the episphere, Erwinia was present the most in the leaf (Table S7).

### Bacterial Community Structure Comparison

**Fig. 2:**
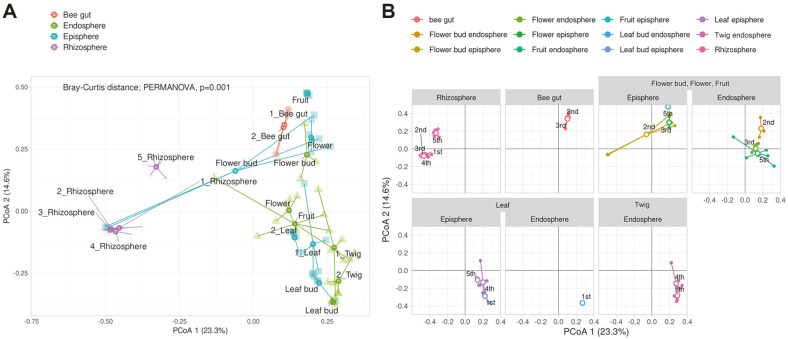
Principal coordinates analysis (PCoA) of Bray-Curtis distances to visualize similarities or dissimilarities between samples. Each point represents the average of the sample and is labeled accordingly. (**A**) Similarity was analyzed across all tissues and stages. Statistical analysis was conducted by permutational analysis of variance (PERMANOVA) and revealed significant differences between samples (*p* = 0.001). Samples were analyzed in 5 replicates in plant tissue and 3 replicates in bee gut. (**B**) Similarity was analyzed within tissues. Samples with the same tissue were grouped and analyzed after development.

The similarities between bacterial communities in different tissues were revealed through Bray-Curtis distance analysis (Fig. 2). The results showed a significance level of *P_adj_* of 0.001, which was confirmed by the permutational analysis of variance (PERMANOVA) and pairwise PERMANOVA as post-hoc tests (Fig. 2A, Table S8). Moreover, Principal Coordinates Analysis (PCoA) revealed that the communities were largely divided into two groups. All rhizospheres were observed to be more similar to each other than to other tissues. The endosphere and episphere showed a relationship with each other, but not with the rhizosphere. Notably, the bee gut, which plays a role in pollination, was found to be related to flower buds in the endosphere and flowers and flower buds in the episphere. To investigate the correlation between bacterial communities and tissues, PCoA and Non-metric Multi-Dimensional Scaling (NMDS) analyses were performed for each tissue (Figs. 2B and S3). In the rhizosphere, the bacterial communities were found to be similar among all five sampling times. Similarities were also observed in flower buds, flowers, and fruits based on their developmental stage. The bee gut, leaves, and twigs showed similar results.

### Bacterial Community Co-Occurrence Network and the Core Taxa in Apple

**Fig. 3:**
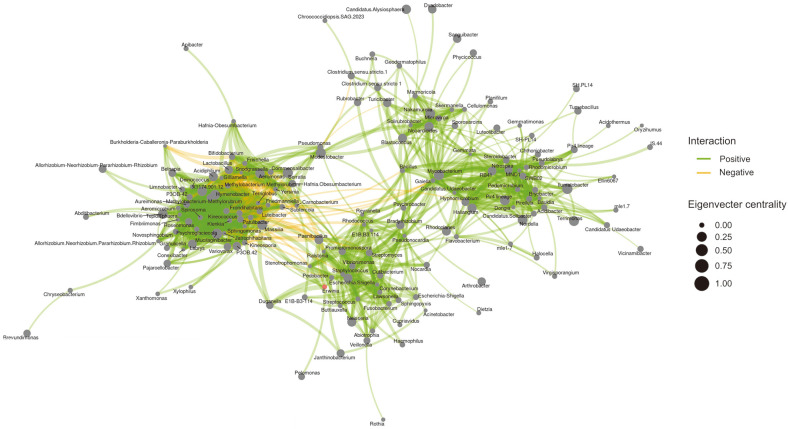
Microbiome co-occurrence network and core taxa of apple. The microbiome co-occurrence network in the apple was constructed using samples excluding the rhizosphere, and correlation values of 0.3 or more were used to represent a positive (green line) or negative (yellow line) relationship between microorganisms. The size of each dot represents its eigen centrality.

Significant interactions were determined using Sparse Correlations for Compositional Data (SparCC) in Amplicon sequence variants (ASVs). We analyzed the network using endosphere and episphere samples and confirmed that it was largely divided into four groups (Fig. 3A). *Ewinia* showed a negative correlation with eight taxa, namely *Acidiphilium*, *Conexibacter*, *Klenkia*, *Labrys*, *Novosphingobium*, *Paenibacillus*, *Pajaroellobacter* and *Terriglobus*. However, the correlation appeared to have low values with a value of approximately -0.2 (Table 1). To determine the core taxa of apples regardless of tissues or seasonal stages, the eigen centrality value was set at a minimum of 0.7 for analysis (Fig. 3B). Results indicated a positive relationship between *Aeromicrobium*, *Aureimonas*, *Burkholderia*, *Caballeronia*, *Paraburkholderia*, *Fimbriimonas*, *Friedmanniella*, *Frondihabitans*, *Hymenobacter*, *Jatrophihabitans*, *Kineococcus*, *Kineosporia*, *Klenkia*, *Luteibacter*, *Mucilaginibacter*, *Patulibacter*, *Psychroglaciecola*, *Roseomonas*, *Tepidisphaera*, *Terriglobus*, *Sphingomonas*, *Spirosoma*, and X1174.901.12. No negative relationships were observed among the 21 taxa, and all of them exhibited a positive correlation. However, *Klenkia* and *Terriglobus* appeared to be frequently associated with negative relationships with *E. amylovora* during core taxa analysis.

### Negative Relationship Taxa against *E. amylovora*

**Fig. 4:**
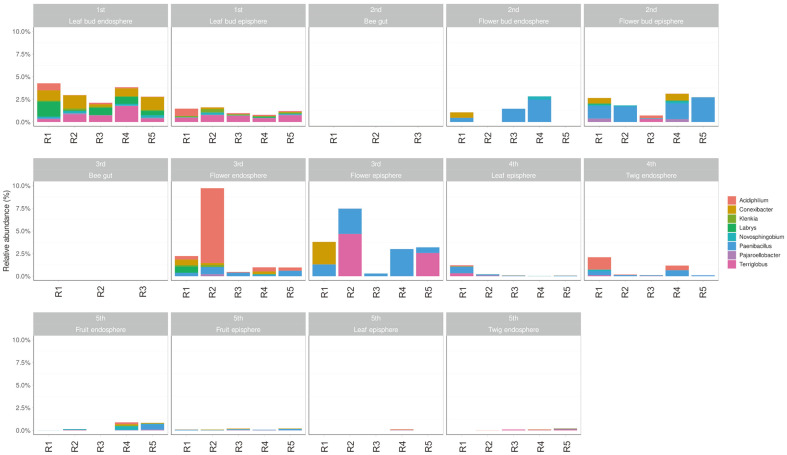
Relative abundance of negative taxa in relation to *E. amylovora* in different tissues and developmental stages of apple. Abundance was analyzed for eight genera showing a negative relationship with *E. amylovora*. The y-axis represents relative abundance, ranging from 0% to 10%.

The relative abundance of eight taxa showing a negative correlation with *E. amylovora* was observed. A total of 8 taxa were present in 16.02% of the endosphere and 6.09% of the episphere at the leaf bud (Fig. 4). During the flower bud period, the sum of the 8 taxa was found to be 5.37% in the endosphere and 11.09% in the episphere. In the flower, the 8 taxa accounted for 14.38% in the endosphere and 17.76% in the episphere. In the twig endosphere, the negative taxa were present in 3.69% and 0.47%, respectively. Similarly, 0.16% and 0.13% of the 8 taxa were present in samplings 4 and 5 of the leaf episphere. In fruit, the negative taxa were present in 0.19% of the endosphere and 0.7% of the episphere. These results indicate that a higher proportion of negative taxa were found in the flower bud and flower, which correlate with the infection periods of *E. amylovora*. In contrast, fewer negative taxa were present in the twig and fruit when *E. amylovora* was not infected, compared to the leaf bud, flower bud, and flower tissues (Table S9).

## Discussion

Apples are an economically important fruit in a modern agricultural society. The area of apple cultivation and production is increasing worldwide, while the variety of and market for processed foods derived from apples are expanding annually, making it a valuable agricultural product that contributes to several industries. During apple cultivation, numerous diseases can occur, including fire blight caused by *E. amylovora*. This disease is considered a major problem because it quickly kills apple trees and causes significant economic losses. Therefore, many countries, such as New Zealand, the United Kingdom, Mexico, and Taiwan have designated *E. amylovora* as a major prohibited pathogen, and when fire blight occurs, all infected apple trees are buried in the soil. Antibiotic-based pesticides are prescribed to control fire blight disease, but the effectiveness of treatment is uncertain. Studies are therefore being conducted to understand fire blight ecology and develop methods for controlling the disease. In this study, we aimed to investigate the bacterial community composition in different developmental stages and tissues of apples to comprehend the structure and shifting of the apple bacterial community, as well as to identify keystone taxa against fire blight disease.

Our results indicated that there were differences in species richness and evenness in the endosphere and episphere depending on the tissues and season, but no differences were observed in the rhizosphere. The endosphere of the flower bud and flower stage exhibited lower richness compared to other tissues [31]. *Burkholderiaceae* comprised a significant portion of the endosphere (2-38%) and endosphere (0.7-75%) in the flower bud and flower stages. This family is known to be part of the core floral community in Arabidopsis [32]. Similar results were observed in apples, highlighting the potential significance of *Burkholderiaceae* in apple flowers and opening up avenues for future research. The epiphytic leaves showed that Erwinia was present among the top 10 most abundant species. However, there was no significant difference in the microbiome of healthy and unhealthy leaves in the episphere as seen in the PCoA analysis [33].

A high correlation between the endosphere and episphere was observed in the PCoA analysis when dividing the groups, as opposed to the rhizosphere. This finding, in conjunction with the importance of the endosphere and episphere in the occurrence of fire blight disease [33], prompted us to conduct a microbiota network analysis. This analysis was performed using the ASV lists of the endosphere and episphere, excluding the rhizosphere. The analysis revealed that eight taxa, namely *Acidiphilium*, *Conexibacter*, *Klenkia*, *Labrys*, *Novosphingobium*, *Paenibacillus*, *Pajaroellobacter*, and *Terriglobus*, showed a negative relationship with *Erwinia amylovora*. These results suggest that these members may play a role as keystone taxa in controlling *E. amylovora*. In particular, the strain *Paenibacillus* polymyxa M-1 has been reported as a suppression agent for the growth of *E. amylovora* [34], and a strain of *Novosphingobium* has been found to effectively control pepper anthracnose [35]. However, the negative relationship taxa had a low correlative value of approximately 0.2, indicating that the control of *E. amylovora* may be achieved through the establishment of a Synthetic Community (SynCom) rather than through the biocontrol of individual microorganisms.

## Supplemental Materials

Supplementary data for this paper are available on-line only at http://jmb.or.kr.

## Figures and Tables

**Table 1:** List of taxa with negative correlation against genus *Ewinia* in SparCC analysis.

Taxa	Correlative	*p*-value
*Acidiphilium*	-0.2186	0.041
*Conexibacter*	-0.2934	0.007
*Klenkia*	-0.2293	0.008
*Labrys*	-0.2114	0.026
*Novosphingobium*	-0.2031	0.039
*Paenibacillus*	-0.3035	0.005
*Pajaroellobacter*	-0.2312	0.017
*Terriglobus*	-0.2280	0.037
