# Supplementary material for: Dynamics of Bacterial Communities by Apple Tissue: Implications for Apple Health
Source: J Microbiol Biotechnol. 2023 Jul 7;33(9):1141–8. doi: 10.4014/jmb.2305.05003 (PMC10580880; doi:10.4014/jmb.2305.05003)
Supplement: Supplementary file 1 [file jmb-33-9-1141-supple.pdf]

J. Microbiol. Biotechnol.
https://doi.org/10.4014/jmb.2305.05003

Supplementary Tables and Figures

Dynamics of Bacterial Communities by Apple Tissues: Implication for Apple Health

Hwajung Lee[1¶], Su-Hyeon Kim[1¶], Da-Ran Kim[2], Gyeongjun Cho[3], Youn-Sig kwak[1,2*]

Table S1. Sampling time and sampling tissues in this study.

Sampling time	Sampling date	Rhizosphere soil	Leaf bud	Flower bud	Flower	Leaf	Twig	fruit	Bee
1	2020. 03. 09	Y*	Y						
2	2020. 04. 09	Y		Y					Y
3	2020. 04. 21	Y			Y				Y
4	2020. 05. 14	Y				Y	Y		
5	2020. 06. 01	Y				Y	Y	Y	

Table S2. Primers used for 16S rRNA amplicon and the blocking PCR in this study.

Primer	Sequences (5'-3')
341F	TCGTCGGCAGCGTCAGATGTGTATAAGAGACAGCCTACGGGNGGCWGCAG
805R	GTCTCGTGGGCTCGGAGATGTGTATAAGAGACAGGACTACHVGGGTATCTAATCC
pPNA	GGCTCAACCCTGGACAG
mPNA	GGCAAGTGTTCTTCGGA

Table S3. Number of sequencings read counts of samples according to tissue and development stage of apple.

Stage	Tissue		Sample name	Replication name	Merged	Nonchim	Tax_bacteria
			B1Rh1	R1	26956	25621	21162
			B1Rh3	R2	34807	33551	25970
	Rhizosphere_1		B1Rh5	R3	28219	27117	23554
			B1Rh7	R4	30721	29320	24883
			B1Rh9	R5	30103	28513	24101
			B1EN1_l	R1	36590	33356	4733
			B1EN3_l	R2	37897	32053	14014
1st	Endosphere	Leaf bud	B1EN5_l	R3	40618	35376	12874
			B1EN7_l	R4	34857	27518	18281
			B1EN9_l	R5	35885	29220	18593
			B1EP1_l	R1	41473	28652	38645
			B1EP3_l	R2	46639	32983	44022
	Episphere	Leaf bud	B1EP5_l	R3	47888	32215	44391
			B1EP7_l	R4	46201	32744	42970
			B1EP9_l	R5	44004	29852	40712
			B2BG1	R1	48001	33588	47051
	Bee gut_1		B2BG2	R2	47010	33956	45495
			B2BG3	R3	51038	37376	50344
			B2Rh1	R1	25906	24680	21074
			B2Rh3	R2	29832	27972	23589
	Rhizosphere_2		B2Rh5	R3	28082	26705	21727
			B2Rh7	R4	28297	27073	23773
			B2Rh9	R5	29755	28615	23554
2nd			B2EN1_f	R1	42041	40417	927
			B2EN3_f	R2	35526	34728	471
	Endosphere	Flower bud	B2EN5_f	R3	37968	37436	625
			B2EN7_f	R4	42584	41576	391
			B2EN9_f	R5	30894	30217	151
			B2EP1_f	R1	33536	32086	26440
			B2EP3_f	R2	49660	41263	17654
	Episphere	Flower bud	B2EP5_f	R3	41364	35253	11066
			B2EP7_f	R4	32433	31082	25609
			B2EP9_f	R5	32223	28811	8939
			B3BG1	R1	52230	41664	50432
	Bee gut_2		B3BG2	R2	38757	26213	37799
			B3BG3	R3	46265	33952	45376
			B3Rh1	R1	29828	28510	23451
			B3Rh3	R2	27465	26044	22251
	Rhizosphere_3		B3Rh5	R3	34501	32943	27628
			B3Rh7	R4	33675	32013	25875
3rd			B3Rh9	R5	25975	24878	20435
			B3EN1_f	R1	39788	38985	2690
			B3EN3_f	R2	37620	37001	2207
	Endosphere	Flower	B3EN5_f	R3	33784	33260	2102
			B3EN7_f	R4	29528	28885	3513
			B3EN9_f	R5	41756	41316	2231
	Episphere	Flower	B3EP1_f	R1	38710	34354	14133
			B3EP3_f	R2	39422	36332	10205

			B3EP5_f	R3	45773	41153	9784
			B3EP7_f	R4	35520	32082	8739
			B3EP9_f	R5	35076	31375	6566
			B4Rh1	R1	36379	34554	27462
			B4Rh3	R2	31468	29751	26102
	Rhizosphere_3		B4Rh5	R3	28496	27164	23349
			B4Rh7	R4	32250	30753	24515
			B4Rh9	R5	29970	28766	22504
			B4EN1_t	R1	40773	40287	1739
			B4EN3_t	R2	43606	42423	2167
4th	Endosphere	Twig	B4EN5_t	R3	46710	41543	17643
			B4EN7_t	R4	41096	40195	1852
			B4EN9_t	R5	40938	39189	3654
			B4EP1_l	R1	30433	26624	10993
			B4EP3_l	R2	33134	26064	21138
	Episphere	Leaf	B4EP5_l	R3	34309	26374	28554
			B4EP7_l	R4	36974	26256	33316
			B4EP9_l	R5	38769	29562	28643
			B5Rh1	R1	57305	50977	28194
			B5Rh3	R2	63007	57105	34352
	Rhizosphere_3		B5Rh5	R3	50782	46043	26613
			B5Rh7	R4	58498	52281	28570
			B5Rh9	R5	50070	44584	27872
			B5EN1_f	R1	48916	46895	10850
			B5EN3_f	R2	48731	47495	6471
	Endosphere	Fruit	B5EN5_f	R3	62233	59897	11103
			B5EN7_f	R4	46924	44721	16972
			B5EN9_f	R5	51139	44978	28359
			B5EN1_t	R1	61896	59036	23126
			B5EN3_t	R2	57956	56009	7980
5th	Endosphere	Twig	B5EN5_t	R3	56603	53659	15803
			B5EN7_t	R4	61176	56397	24154
			B5EN9_t	R5	58330	57234	3562
			B5EP1_f	R1	49832	40323	28585
			B5EP3_f	R2	61559	48424	32663
	Episphere	Fruit	B5EP5_f	R3	56772	45411	30482
			B5EP7_f	R4	58771	46359	31412
			B5EP9_f	R5	59870	47539	33048
			B5EP1_l	R1	57012	42605	51230
			B5EP3_l	R2	58182	43258	52964
	Episphere	Leaf	B5EP5_l	R3	61627	46358	54604
			B5EP7_l	R4	65252	53164	62258
			B5EP9_l	R5	63170	47798	55187

Table S4. GenBank accession numbers in this study.

Sample	BioProject	BioSample	SRA
B1Rh1	PRJNA905917	SAMN31887055	SRR22425797
B1Rh3	PRJNA905917	SAMN31887074	SRR22425796
B1Rh5	PRJNA905917	SAMN31887075	SRR22425795
B1Rh7	PRJNA905917	SAMN31887077	SRR22425794
B1Rh9	PRJNA905917	SAMN31887078	SRR22425793
B1EN1_l	PRJNA905917	SAMN31887079	SRR22438480
B1EN3_l	PRJNA905917	SAMN31887080	SRR22438479
B1EN5_l	PRJNA905917	SAMN31887081	SRR22438478
B1EN7_l	PRJNA905917	SAMN31887082	SRR22438477
B1EN9_l	PRJNA905917	SAMN31887083	SRR22438476
B1EP1_l	PRJNA905917	SAMN31887084	SRR22438486
B1EP3_l	PRJNA905917	SAMN31887085	SRR22438485
B1EP5_l	PRJNA905917	SAMN31887086	SRR22438484
B1EP7_l	PRJNA905917	SAMN31887087	SRR22438483
B1EP9_l	PRJNA905917	SAMN31887088	SRR22438482
B2Rh1	PRJNA905917	SAMN31887111	SRR22438491
B2Rh3	PRJNA905917	SAMN31887112	SRR22438490
B2Rh5	PRJNA905917	SAMN31887113	SRR22438489
B2Rh7	PRJNA905917	SAMN31887114	SRR22438488
B2Rh9	PRJNA905917	SAMN31887337	SRR22438487
B2EN1_f	PRJNA905917	SAMN31887353	SRR22438496
B2EN3_f	PRJNA905917	SAMN31887354	SRR22438495
B2EN5_f	PRJNA905917	SAMN31887355	SRR22438494
B2EN7_f	PRJNA905917	SAMN31887356	SRR22438493
B2EN9_f	PRJNA905917	SAMN31887357	SRR22438492
B2EP1_f	PRJNA905917	SAMN31887358	SRR22438501
B2EP3_f	PRJNA905917	SAMN31887365	SRR22438500
B2EP5_f	PRJNA905917	SAMN31887366	SRR22438499
B2EP7_f	PRJNA905917	SAMN31887367	SRR22438498
B2EP9_f	PRJNA905917	SAMN31887368	SRR22438497
B2BG1	PRJNA905917	SAMN31887369	SRR22438504
B2BG2	PRJNA905917	SAMN31887370	SRR22438503
B2BG3	PRJNA905917	SAMN31887377	SRR22438502
B3Rh1	PRJNA905917	SAMN31887378	SRR22438509
B3Rh3	PRJNA905917	SAMN31887379	SRR22438508
B3Rh5	PRJNA905917	SAMN31887380	SRR22438507
B3Rh7	PRJNA905917	SAMN31887381	SRR22438506
B3Rh9	PRJNA905917	SAMN31887382	SRR22438505
B3EN1_f	PRJNA905917	SAMN31887384	SRR22438514
B3EN3_f	PRJNA905917	SAMN31887385	SRR22438513
B3EN5_f	PRJNA905917	SAMN31887386	SRR22438512
B3EN7_f	PRJNA905917	SAMN31887387	SRR22438511
B3EN9_f	PRJNA905917	SAMN31887388	SRR22438510
B3EP1_f	PRJNA905917	SAMN31887389	SRR22438519
B3EP3_f	PRJNA905917	SAMN31887474	SRR22438518
B3EP5_f	PRJNA905917	SAMN31887475	SRR22438517
B3EP7_f	PRJNA905917	SAMN31887476	SRR22438516
B3EP9_f	PRJNA905917	SAMN31887477	SRR22438515
B3BG1	PRJNA905917	SAMN31887478	SRR22438527
B3BG2	PRJNA905917	SAMN31887479	SRR22438526
B3BG3	PRJNA905917	SAMN31887480	SRR22438525
B4Rh1	PRJNA905917	SAMN31887642	SRR22438532
B4Rh3	PRJNA905917	SAMN31887644	SRR22438531

B4Rh5	PRJNA905917	SAMN31887646	SRR22438530
B4Rh7	PRJNA905917	SAMN31887647	SRR22438529
B4Rh9	PRJNA905917	SAMN31887648	SRR22438528
B4EN1_t	PRJNA905917	SAMN31887652	SRR22439037
B4EN3_t	PRJNA905917	SAMN31887653	SRR22439036
B4EN5_t	PRJNA905917	SAMN31887659	SRR22439035
B4EN7_t	PRJNA905917	SAMN31887660	SRR22439034
B4EN9_t	PRJNA905917	SAMN31887661	SRR22439033
B4EP1_l	PRJNA905917	SAMN31887662	SRR22439114
B4EP3_l	PRJNA905917	SAMN31888060	SRR22439113
B4EP5_l	PRJNA905917	SAMN31888070	SRR22439112
B4EP7_l	PRJNA905917	SAMN31888071	SRR22439111
B4EP9_l	PRJNA905917	SAMN31888073	SRR22439110
B5Rh1	PRJNA721544	SAMN18720833	SRR14277116
B5Rh3	PRJNA721544	SAMN18744516	SRR14277115
B5Rh5	PRJNA721544	SAMN18744520	SRR14277114
B5Rh7	PRJNA721544	SAMN18744521	SRR14277113
B5Rh9	PRJNA721544	SAMN18745231	SRR14277112
B5EN1_t	PRJNA722110	SAMN18745293	SRR14278204
B5EN3_t	PRJNA722110	SAMN18745906	SRR14278203
B5EN5_t	PRJNA722110	SAMN18745907	SRR14278202
B5EN7_t	PRJNA722110	SAMN18745962	SRR14278201
B5EN9_t	PRJNA722110	SAMN18745969	SRR14278200
B5EN1_f	PRJNA722126	SAMN18746006	SRR14278210
B5EN3_f	PRJNA722126	SAMN18746008	SRR14278209
B5EN5_f	PRJNA722126	SAMN18746102	SRR14278208
B5EN7_f	PRJNA722126	SAMN18746109	SRR14278207
B5EN9_f	PRJNA722126	SAMN18746110	SRR14278206
B5EP1_f	PRJNA722134	SAMN18746111	SRR14278328
B5EP3_f	PRJNA722134	SAMN18746112	SRR14278327
B5EP5_f	PRJNA722134	SAMN18746144	SRR14278326
B5EP7_f	PRJNA722126	SAMN18746145	SRR14278325
B5EP9_f	PRJNA722134	SAMN18746803	SRR14278324
B5EP1_l	PRJNA722161	SAMN18746822	SRR14278390
B5EP3_l	PRJNA722161	SAMN18746831	SRR14278389
B5EP5_l	PRJNA722161	SAMN18746855	SRR14278388
B5EP7_l	PRJNA722161	SAMN18746911	SRR14278387
B5EP9_l	PRJNA722161	SAMN18746918	SRR14278386

Table S5. Genus level list of top 10 ASV with high relative abundance in rhizosphere.

	1st	2nd	3rd	4th	5th
1	*Pseudomonas* (ASV_27)	*Pseudolabrys* (ASV_33)	*Hyphomicrobium* (ASV_36)	*Hyphomicrobium* (ASV_36)	*Pseudomonas* (ASV_1)
2	*Hyphomicrobium* (ASV_38)	*Hyphomicrobium* (ASV_36)	*Hyphomicrobium* (ASV_38)	*Skermanella* (ASV_57)	*Streptomyces* (ASV_6)
3	*Hyphomicrobium* (ASV_36)	*Pseudomonas* (ASV_27)	*Pseudolabrys* (ASV_33)	*Pseudolabrys* (ASV_33)	*Pseudomonas* (ASV_27)
4	*Pseudolabrys* (ASV_33)	*Pseudomonas* (ASV_109)	*Gaiella* (ASV_47)	*Hyphomicrobium* (ASV_38)	*Ralstonia* (ASV_3)
5	*Gaiella* (ASV_47)	*Hyphomicrobium* (ASV_38)	*Skermanella* (ASV_57)	*Dongia* (ASV_55)	*Escherichia-Shigella* (ASV_15)
6	*Pseudomonas* (ASV_131)	Candidatus *Udaeobacter* (ASV_62)	*Gaiella* (ASV_60)	*Gaiella* (ASV_47)	*Pseudomonas* (ASV_14)
7	Candidatus *Koribacter* (ASV_72)	*Gaiella* (ASV_54)	*Nitrospira* (ASV_53)	*Nitrospira* (ASV_53)	*Pseudolabrys* (ASV_33)
8	*Nitrospira* (ASV_53)	*Nitrospira* (ASV_53)	*Dongia* (ASV_55)	*IS-44* (ASV_68)	*Hyphomicrobium* (ASV_38)
9	*IS-44* (ASV_68)	*Gaiella* (ASV_47)	*Gaiella* (ASV_54)	*Gaiella* (ASV_60)	*Hyphomicrobium* (ASV_36)
10	*Gaiella* (ASV_54)	*Hyphomicrobium* (ASV_77)	Candidatus *Udaeobacter* (ASV_80)	Candidatus *Xiphinematobacter* (ASV_151)	*Gaiella* (ASV_47)

Table S6. Genus level list of top 10 ASV with high relative abundance in endosphere.

	Flower bud	Flower	Fruit
1	*Pseudomonas* (ASV_1)	*Ralstonia* (ASV_3)	*Ralstonia* (ASV_3)
2	*Ralstonia* (ASV_3)	*Bifidobacterium* (ASV_67)	*Pseudomonas* (ASV_2)
3	*Escherichia-Shigella* (ASV_15)	*Lactobacillus* (ASV_52)	*Erwinia* (ASV_4)
4	*Lactobacillus* (ASV_423)	*Methylobacterium-Methylorubrum* (ASV_9)	*Erwinia* (ASV_5)
5	*Patulibacter* (ASV_71)	*Lactobacillus* (ASV_326)	*Sphingomonas* (ASV_22)
6	*Stenotrophomonas* (ASV_40)	*Skermanella* (ASV_57)	*Pseudomonas* (ASV_17)
7	*Streptomyces* (ASV_6)	*Buchnera* (ASV_227)	*Pseudomonas* (ASV_21)
8	*Lactobacillus* (ASV_290)	*Lactobacillus* (ASV_177)	*Massilia* (ASV_20)
9	*Gilliamella* (ASV_159)	*Acidiphilium* (ASV_639)	*Rhodococcus* (ASV_94)
10	*Craurococcus-Caldovatus* (ASV_4279)	*Burkholderia-Caballeronia-Paraburkholderia* (ASV_26)	*Pseudomonas* (ASV_12)
	Leaf bud	Twig_1	Twig_2
1	*Pseudomonas* (ASV_2)	*Pseudomonas* (ASV_2)	*Pseudomonas* (ASV_2)
2	*Methylobacterium-Methylorubrum* (ASV_9)	*Ralstonia* (ASV_3)	*Pseudomonas* (ASV_17)
3	*Burkholderia-Caballeronia-Paraburkholderia* (ASV_26)	*Massilia* (ASV_20)	*Methylobacterium-Methylorubrum* (ASV_9)
4	*Frondihabitans* (ASV_71)	*Pseudomonas* (ASV_7)	*Ralstonia* (ASV_3)
5	*Kineococcus* (ASV_25)	*Pseudomonas* (ASV_8)	*Frondihabitans* (ASV_16)
6	*1174-901-12* (ASV_23)	*Massilia* (ASV_117)	*Pseudomonas* (ASV_7)
7	*Patulibacter* (ASV_71)	*Methylobacterium-Methylorubrum* (ASV_9)	*Pseudomonas* (ASV_8)
8	*Pseudomonas* (ASV_17)	*Ralstonia* (ASV_63)	*Pseudomonas* (ASV_29)
9	*Psychroglaciecola* (ASV_115)	*Pseudomonas* (ASV_29)	*Massilia* (ASV_20)
10	*Kineococcus* (ASV_92)	*Burkholderia-Caballeronia-Paraburkholderia*(ASV_979)	*Pseudomonas* (ASV_48)

Table S7. Genus level list of top 10 ASV with high relative abundance in episphere.

	Flower bud	Flower	Fruit
1	*Ralstonia* (ASV_3)	*Ralstonia* (ASV_3)	*Pseudomonas* (ASV_1)
2	*Pseudomonas* (ASV_1)	*Pseudomonas* (ASV_1)	*Streptomyces* (ASV_6)
3	*Pseudomonas* (ASV_10)	*Stenotrophomonas* (ASV_40)	*Ralstonia* (ASV_3)
4	*Escherichia-Shigella* (ASV_15)	*Streptomyces* (ASV_6)	*Escherichia-Shigella* (ASV_15)
5	*Methylobacterium-Methylorubrum* (ASV_9)	*Rhodococcus* (ASV_37)	*Pseudomonas* (ASV_14)
6	*Rhodococcus* (ASV_37)	*Burkholderia-Caballeronia-Paraburkholderia* (ASV_76)	*Pseudonocardia* (ASV_32)
7	*Stenotrophomonas* (ASV_40)	*Terriglobus* (ASV_134)	*Staphylococcus* (ASV_50)
8	*Ralstonia* (ASV_379)	*Lactobacillus* (ASV_193)	*Pseudomonas* (ASV_24)
9	*Streptomyces* (ASV_422)	*Ralstonia* (ASV_426)	*Streptomyces* (ASV_81)
10	*1174-901-12* (ASV_23)	*Paenibacillus* (ASV_165)	*Promicromonospora* (ASV_66)
	Leaf bud	Leaf_1	Leaf_2
1	*Methylobacterium-Methylorubrum* (ASV_9)	*Pseudomonas* (ASV_2)	*Erwinia* (ASV_4)
2	*Hymenobacter* (ASV_18)	*Pseudomonas* (ASV_7)	*Erwinia* (ASV_5)
3	*Frondihabitans* (ASV_16)	*Pseudomonas* (ASV_8)	*Pseudomonas* (ASV_13)
4	*Pseudomonas* (ASV_2)	*Ralstonia* (ASV_2)	*Pseudomonas* (ASV_7)
5	*1174-901-12* (ASV_23)	*Pseudomonas* (ASV_39)	*Pseudomonas* (ASV_8)
6	*Hymenobacter* (ASV_30)	*Pseudomonas* (ASV_49)	*Pseudomonas* (ASV_19)
7	*Hymenobacter* (ASV_41)	*Pseudomonas* (ASV_35)	*Pseudomonas* (ASV_2)
8	*Hymenobacter* (ASV_45)	*Pseudomonas* (ASV_73)	*Pseudomonas* (ASV_21)
9	*Sphingomonas* (ASV_34)	*Pseudomonas* (ASV_14)	*Sphingomonas* (ASV_22)
10	*Kineococcus* (ASV_25)	*Pseudomonas* (ASV_64)	*Ralstonia* (ASV_3)

Table S8. Genus level list of top 10 ASV with high relative abundance in bee gut.

	Bee gut in 2th	Bee gut in 2th
1	*Pseudomonas* (ASV_1)	*Pseudomonas* (ASV_1)
2	*Pseudomonas* (ASV_10)	*Pseudomonas* (ASV_11)
3	*Pseudomonas* (ASV_14)	*Pseudomonas* (ASV_12)
4	*Pseudomonas* (ASV_24)	*Yersinia* (ASV 28)
5	*Pseudomonas* (ASV_12)	*Aeromonas* (ASV_31)
6	*Pseudomonas* (ASV 11)	*Yersinia* (ASV_46)
7	*Pseudomonas* (ASV 44)	*Pseudomonas* (ASV_10)
8	*Pseudomonas* (ASV 43)	*Pseudomonas* (ASV_43)
9	*Aeromonas* (ASV 31)	*Aeromonas* (ASV_192)
10	*Yersinia* (ASV 46)	*Hafnia-Obesumbacteriums* (ASV_150)

12 Table S9. Pairwise PERMANOVA as post-hoc with FDR method (*:P_{adj}> 0.05).

Compare group in pairwise PERMANOVA	degrees of freedom	Sums of squares	Model F-statistics	R^2	*P* value	P_{adj} (FDR method)	
Leaf bud endosphere 1st vs Leaf bud episphere 1st	1	0.0024	18.9867	0.7036	0.0130	0.0232	*
Leaf bud endosphere 1st vs Rhizosphere 1st	1	0.0489	248.2367	0.9688	0.0060	0.0228	*
Leaf bud endosphere 1st vs bee gut 2nd	1	0.0189	92.0840	0.9388	0.0180	0.0259	*
Leaf bud endosphere 1st vs Flower bud endosphere 2nd	1	0.0125	25.8343	0.8115	0.0180	0.0259	*
Leaf bud endosphere 1st vs Flower bud episphere 2nd	1	0.0235	6.0498	0.4306	0.0090	0.0228	*
Leaf bud endosphere 1st vs Rhizosphere 2nd	1	0.0529	413.9186	0.9810	0.0080	0.0228	*
Leaf bud endosphere 1st vs bee gut 3rd	1	0.0180	112.5635	0.9494	0.0180	0.0259	*
Leaf bud endosphere 1st vs Flower endosphere 3rd	1	0.0109	28.5212	0.7809	0.0070	0.0228	*
Leaf bud endosphere 1st vs Flower episphere 3rd	1	0.0230	119.4366	0.9372	0.0070	0.0228	*
Leaf bud endosphere 1st vs Rhizosphere 3rd	1	0.0537	401.4427	0.9805	0.0130	0.0232	*
Leaf bud endosphere 1st vs Leaf episphere 4th	1	0.0073	11.4224	0.5881	0.0080	0.0228	*
Leaf bud endosphere 1st vs New twigs endosphere 4th	1	0.0083	13.9209	0.6351	0.0090	0.0228	*
Leaf bud endosphere 1st vs Rhizosphere 4th	1	0.0507	317.6815	0.9754	0.0100	0.0228	*
Leaf bud endosphere 1st vs Fruit endosphere 5th	1	0.0046	11.3482	0.5865	0.0050	0.0228	*
Leaf bud endosphere 1st vs Fruit episphere 5th	1	0.0127	9.6321	0.5463	0.0080	0.0228	*
Leaf bud endosphere 1st vs Leaf episphere 5th	1	0.0383	815.8006	0.9903	0.0140	0.0237	*
Leaf bud endosphere 1st vs New twigs endosphere 5th	1	0.0120	37.9756	0.8260	0.0080	0.0228	*
Leaf bud endosphere 1st vs Rhizosphere 5th	1	0.0582	284.8861	0.9727	0.0050	0.0228	*
Leaf bud episphere 1st vs Rhizosphere 1st	1	0.0405	174.1573	0.9561	0.0060	0.0228	*
Leaf bud episphere 1st vs bee gut 2nd	1	0.0143	56.5241	0.9040	0.0130	0.0232	*
Leaf bud episphere 1st vs Flower bud endosphere 2nd	1	0.0097	18.3540	0.7536	0.0170	0.0257	*
Leaf bud episphere 1st vs Flower bud episphere 2nd	1	0.0178	4.5538	0.3627	0.0130	0.0232	*
Leaf bud episphere 1st vs Rhizosphere 2nd	1	0.0442	270.2226	0.9712	0.0090	0.0228	*
Leaf bud episphere 1st vs bee gut 3rd	1	0.0136	65.2312	0.9158	0.0210	0.0272	*
Leaf bud episphere 1st vs Flower endosphere 3rd	1	0.0086	20.7085	0.7213	0.0090	0.0228	*
Leaf bud episphere 1st vs Flower episphere 3rd	1	0.0190	83.2851	0.9124	0.0110	0.0228	*
Leaf bud episphere 1st vs Rhizosphere 3rd	1	0.0449	264.5732	0.9707	0.0060	0.0228	*
Leaf bud episphere 1st vs Leaf episphere 4th	1	0.0093	13.7303	0.6319	0.0080	0.0228	*
Leaf bud episphere 1st vs New twigs endosphere 4th	1	0.0091	14.3925	0.6427	0.0080	0.0228	*
Leaf bud episphere 1st vs Rhizosphere 4th	1	0.0422	215.9353	0.9643	0.0050	0.0228	*
Leaf bud episphere 1st vs Fruit endosphere 5th	1	0.0073	16.5247	0.6738	0.0090	0.0228	*
Leaf bud episphere 1st vs Fruit episphere 5th	1	0.0127	9.3769	0.5396	0.0080	0.0228	*
Leaf bud episphere 1st vs Leaf episphere 5th	1	0.0331	400.7843	0.9804	0.0070	0.0228	*
Leaf bud episphere 1st vs New twigs endosphere 5th	1	0.0094	26.7553	0.7698	0.0110	0.0228	*

Leaf bud episphere 1st vs Rhizosphere 5th	1	0.0489	203.5792	0.9622	0.0110	0.0228	*
Rhizosphere 1st vs bee gut 2nd	1	0.0248	71.2138	0.9223	0.0260	0.0320	*
Rhizosphere 1st vs Flower bud endosphere 2nd	1	0.0290	46.3645	0.8854	0.0200	0.0271	*
Rhizosphere 1st vs Flower bud episphere 2nd	1	0.0177	4.4461	0.3572	0.1420	0.1619	
Rhizosphere 1st vs Rhizosphere 2nd	1	0.0002	0.8787	0.0990	0.5150	0.5180	
Rhizosphere 1st vs bee gut 3rd	1	0.0237	78.1004	0.9287	0.0210	0.0272	*
Rhizosphere 1st vs Flower endosphere 3rd	1	0.0299	61.3116	0.8846	0.0080	0.0228	*
Rhizosphere 1st vs Flower episphere 3rd	1	0.0430	143.5032	0.9472	0.0110	0.0228	*
Rhizosphere 1st vs Rhizosphere 3rd	1	0.0004	1.6935	0.1747	0.1180	0.1373	
Rhizosphere 1st vs Leaf episphere 4th	1	0.0499	66.7231	0.8929	0.0110	0.0228	*
Rhizosphere 1st vs New twigs endosphere 4th	1	0.0382	53.9457	0.8709	0.0100	0.0228	*
Rhizosphere 1st vs Rhizosphere 4th	1	0.0004	1.5384	0.1613	0.1550	0.1710	
Rhizosphere 1st vs Fruit endosphere 5th	1	0.0520	101.7048	0.9271	0.0120	0.0228	*
Rhizosphere 1st vs Fruit episphere 5th	1	0.0353	24.7135	0.7555	0.0070	0.0228	*
Rhizosphere 1st vs Leaf episphere 5th	1	0.0556	359.2582	0.9782	0.0090	0.0228	*
Rhizosphere 1st vs New twigs endosphere 5th	1	0.0317	74.8935	0.9035	0.0080	0.0228	*
Rhizosphere 1st vs Rhizosphere 5th	1	0.0071	22.8246	0.7405	0.0010	0.0228	*
bee gut 2nd vs Flower bud endosphere 2nd	1	0.0034	4.0316	0.5020	0.2000	0.2151	
bee gut 2nd vs Flower bud episphere 2nd	1	0.0067	1.2659	0.1742	0.3090	0.3262	
bee gut 2nd vs Rhizosphere 2nd	1	0.0275	107.1888	0.9470	0.0260	0.0320	*
bee gut 2nd vs bee gut 3rd	1	0.0001	0.2911	0.0678	0.5000	0.5059	
bee gut 2nd vs Flower endosphere 3rd	1	0.0087	14.7185	0.7104	0.0210	0.0272	*
bee gut 2nd vs Flower episphere 3rd	1	0.0072	21.1075	0.7787	0.0140	0.0237	*
bee gut 2nd vs Rhizosphere 3rd	1	0.0283	107.0756	0.9469	0.0170	0.0257	*
bee gut 2nd vs Leaf episphere 4th	1	0.0150	15.9175	0.7262	0.0200	0.0271	*
bee gut 2nd vs New twigs endosphere 4th	1	0.0115	13.0339	0.6848	0.0180	0.0259	*
bee gut 2nd vs Rhizosphere 4th	1	0.0271	90.7108	0.9380	0.0190	0.0266	*
bee gut 2nd vs Fruit endosphere 5th	1	0.0182	29.1382	0.8292	0.0190	0.0266	*
bee gut 2nd vs Fruit episphere 5th	1	0.0123	6.6265	0.5248	0.0230	0.0289	*
bee gut 2nd vs Leaf episphere 5th	1	0.0082	55.2741	0.9021	0.0140	0.0237	*
bee gut 2nd vs New twigs endosphere 5th	1	0.0104	20.4452	0.7731	0.0170	0.0257	*
bee gut 2nd vs Rhizosphere 5th	1	0.0207	57.6325	0.9057	0.0200	0.0271	*
Flower bud endosphere 2nd vs Flower bud episphere 2nd	1	0.0040	0.7237	0.1076	0.4690	0.4774	
Flower bud endosphere 2nd vs Rhizosphere 2nd	1	0.0318	59.5049	0.9084	0.0150	0.0240	*
Flower bud endosphere 2nd vs bee gut 3rd	1	0.0031	3.9982	0.4999	0.2000	0.2151	
Flower bud endosphere 2nd vs Flower endosphere 3rd	1	0.0023	2.6315	0.3049	0.0380	0.0458	*
Flower bud endosphere 2nd vs Flower episphere 3rd	1	0.0015	2.4008	0.2858	0.0330	0.0403	*

Flower bud endosphere 2nd vs Rhizosphere 3rd	1	0.0323	59.5473	0.9085	0.0120	0.0228	*
Flower bud endosphere 2nd vs Leaf episphere 4th	1	0.0061	4.9870	0.4539	0.0160	0.0253	*
Flower bud endosphere 2nd vs New twigs endosphere 4th	1	0.0057	4.8948	0.4493	0.0390	0.0466	*
Flower bud endosphere 2nd vs Rhizosphere 4th	1	0.0302	52.4962	0.8974	0.0220	0.0283	*
Flower bud endosphere 2nd vs Fruit endosphere 5th	1	0.0105	11.6523	0.6601	0.0190	0.0266	*
Flower bud endosphere 2nd vs Fruit episphere 5th	1	0.0039	1.8222	0.2330	0.1110	0.1300	
Flower bud endosphere 2nd vs Leaf episphere 5th	1	0.0051	12.0680	0.6679	0.0210	0.0272	*
Flower bud endosphere 2nd vs New twigs endosphere 5th	1	0.0077	9.8450	0.6213	0.0110	0.0228	*
Flower bud endosphere 2nd vs Rhizosphere 5th	1	0.0200	31.3815	0.8395	0.0240	0.0300	*
Flower bud episphere 2nd vs Rhizosphere 2nd	1	0.0201	5.1302	0.3907	0.1480	0.1676	
Flower bud episphere 2nd vs bee gut 3rd	1	0.0060	1.1544	0.1614	0.3480	0.3629	
Flower bud episphere 2nd vs Flower endosphere 3rd	1	0.0044	1.0522	0.1162	0.3810	0.3925	
Flower bud episphere 2nd vs Flower episphere 3rd	1	0.0060	1.5170	0.1594	0.4470	0.4577	
Flower bud episphere 2nd vs Rhizosphere 3rd	1	0.0207	5.2621	0.3968	0.1300	0.1492	
Flower bud episphere 2nd vs Leaf episphere 4th	1	0.0162	3.6501	0.3133	0.0060	0.0228	*
Flower bud episphere 2nd vs New twigs endosphere 4th	1	0.0117	2.6713	0.2503	0.0530	0.0629	
Flower bud episphere 2nd vs Rhizosphere 4th	1	0.0189	4.7761	0.3738	0.1570	0.1710	
Flower bud episphere 2nd vs Fruit endosphere 5th	1	0.0222	5.2942	0.3982	0.0080	0.0228	*
Flower bud episphere 2nd vs Fruit episphere 5th	1	0.0081	1.5812	0.1650	0.2810	0.2985	
Flower bud episphere 2nd vs Leaf episphere 5th	1	0.0149	3.8733	0.3262	0.0090	0.0228	*
Flower bud episphere 2nd vs New twigs endosphere 5th	1	0.0127	3.0982	0.2792	0.0940	0.1109	
Flower bud episphere 2nd vs Rhizosphere 5th	1	0.0088	2.1991	0.2156	0.1550	0.1710	
Rhizosphere 2nd vs bee gut 3rd	1	0.0262	124.0259	0.9539	0.0180	0.0259	*
Rhizosphere 2nd vs Flower endosphere 3rd	1	0.0330	78.8605	0.9079	0.0030	0.0228	*
Rhizosphere 2nd vs Flower episphere 3rd	1	0.0469	203.1241	0.9621	0.0130	0.0232	*
Rhizosphere 2nd vs Rhizosphere 3rd	1	0.0002	1.0256	0.1136	0.3780	0.3917	
Rhizosphere 2nd vs Leaf episphere 4th	1	0.0541	79.6583	0.9087	0.0130	0.0232	*
Rhizosphere 2nd vs New twigs endosphere 4th	1	0.0418	65.5224	0.8912	0.0120	0.0228	*
Rhizosphere 2nd vs Rhizosphere 4th	1	0.0003	1.3095	0.1407	0.2390	0.2554	
Rhizosphere 2nd vs Fruit endosphere 5th	1	0.0562	127.0245	0.9408	0.0120	0.0228	*
Rhizosphere 2nd vs Fruit episphere 5th	1	0.0388	28.5025	0.7808	0.0150	0.0240	*
Rhizosphere 2nd vs Leaf episphere 5th	1	0.0596	697.3237	0.9887	0.0070	0.0228	*
Rhizosphere 2nd vs New twigs endosphere 5th	1	0.0351	99.0984	0.9253	0.0170	0.0257	*
Rhizosphere 2nd vs Rhizosphere 5th	1	0.0079	32.4076	0.8020	0.0090	0.0228	*
bee gut 3rd vs Flower endosphere 3rd	1	0.0080	14.6754	0.7098	0.0230	0.0289	*
bee gut 3rd vs Flower episphere 3rd	1	0.0067	22.6441	0.7905	0.0090	0.0228	*
bee gut 3rd vs Rhizosphere 3rd	1	0.0270	123.1946	0.9536	0.0210	0.0272	*

bee gut 3rd vs Leaf episphere 4th	1	0.0141	15.7366	0.7240	0.0150	0.0240	*
bee gut 3rd vs New twigs endosphere 4th	1	0.0109	12.9852	0.6840	0.0180	0.0259	*
bee gut 3rd vs Rhizosphere 4th	1	0.0259	102.1534	0.9445	0.0210	0.0272	*
bee gut 3rd vs Fruit endosphere 5th	1	0.0173	29.8735	0.8327	0.0200	0.0271	*
bee gut 3rd vs Fruit episphere 5th	1	0.0114	6.3298	0.5134	0.0150	0.0240	*
bee gut 3rd vs Leaf episphere 5th	1	0.0087	83.8378	0.9332	0.0120	0.0228	*
bee gut 3rd vs New twigs endosphere 5th	1	0.0099	21.4096	0.7811	0.0230	0.0289	*
bee gut 3rd vs Rhizosphere 5th	1	0.0198	63.0563	0.9131	0.0170	0.0257	*
Flower endosphere 3rd vs Flower episphere 3rd	1	0.0061	12.5997	0.6116	0.0090	0.0228	*
Flower endosphere 3rd vs Rhizosphere 3rd	1	0.0335	78.9071	0.9079	0.0120	0.0228	*
Flower endosphere 3rd vs Leaf episphere 4th	1	0.0049	5.2634	0.3968	0.0150	0.0240	*
Flower endosphere 3rd vs New twigs endosphere 4th	1	0.0043	4.8395	0.3769	0.0370	0.0449	*
Flower endosphere 3rd vs Rhizosphere 4th	1	0.0311	69.0801	0.8962	0.0060	0.0228	*
Flower endosphere 3rd vs Fruit endosphere 5th	1	0.0092	13.1878	0.6224	0.0090	0.0228	*
Flower endosphere 3rd vs Fruit episphere 5th	1	0.0027	1.6799	0.1735	0.1500	0.1688	
Flower endosphere 3rd vs Leaf episphere 5th	1	0.0156	46.1876	0.8524	0.0080	0.0228	*
Flower endosphere 3rd vs New twigs endosphere 5th	1	0.0080	13.1977	0.6226	0.0110	0.0228	*
Flower endosphere 3rd vs Rhizosphere 5th	1	0.0267	53.9569	0.8709	0.0100	0.0228	*
Flower episphere 3rd vs Rhizosphere 3rd	1	0.0476	201.0069	0.9617	0.0140	0.0237	*
Flower episphere 3rd vs Leaf episphere 4th	1	0.0113	15.1414	0.6543	0.0060	0.0228	*
Flower episphere 3rd vs New twigs endosphere 4th	1	0.0115	16.3231	0.6711	0.0100	0.0228	*
Flower episphere 3rd vs Rhizosphere 4th	1	0.0449	171.1095	0.9553	0.0090	0.0228	*
Flower episphere 3rd vs Fruit endosphere 5th	1	0.0190	37.5562	0.8244	0.0070	0.0228	*
Flower episphere 3rd vs Fruit episphere 5th	1	0.0079	5.5430	0.4093	0.0080	0.0228	*
Flower episphere 3rd vs Leaf episphere 5th	1	0.0075	50.0217	0.8621	0.0110	0.0228	*
Flower episphere 3rd vs New twigs endosphere 5th	1	0.0147	35.1816	0.8147	0.0090	0.0228	*
Flower episphere 3rd vs Rhizosphere 5th	1	0.0291	94.5366	0.9220	0.0080	0.0228	*
Rhizosphere 3rd vs Leaf episphere 4th	1	0.0548	80.0528	0.9091	0.0090	0.0228	*
Rhizosphere 3rd vs New twigs endosphere 4th	1	0.0427	66.2558	0.8923	0.0090	0.0228	*
Rhizosphere 3rd vs Rhizosphere 4th	1	0.0001	0.5588	0.0653	0.7770	0.7770	
Rhizosphere 3rd vs Fruit endosphere 5th	1	0.0570	127.2540	0.9409	0.0060	0.0228	*
Rhizosphere 3rd vs Fruit episphere 5th	1	0.0394	28.8217	0.7827	0.0080	0.0228	*
Rhizosphere 3rd vs Leaf episphere 5th	1	0.0606	662.3027	0.9881	0.0100	0.0228	*
Rhizosphere 3rd vs New twigs endosphere 5th	1	0.0360	100.1083	0.9260	0.0070	0.0228	*
Rhizosphere 3rd vs Rhizosphere 5th	1	0.0083	33.2741	0.8062	0.0080	0.0228	*
Leaf episphere 4th vs New twigs endosphere 4th	1	0.0016	1.3669	0.1459	0.3110	0.3263	
Leaf episphere 4th vs Rhizosphere 4th	1	0.0517	72.7179	0.9009	0.0040	0.0228	*

Leaf episphere 4th vs Fruit endosphere 5th	1	0.0016	1.6637	0.1722	0.1560	0.1710	
Leaf episphere 4th vs Fruit episphere 5th	1	0.0033	1.7838	0.1823	0.1240	0.1433	
Leaf episphere 4th vs Leaf episphere 5th	1	0.0241	40.2927	0.8343	0.0070	0.0228	*
Leaf episphere 4th vs New twigs endosphere 5th	1	0.0111	12.7727	0.6149	0.0080	0.0228	*
Leaf episphere 4th vs Rhizosphere 5th	1	0.0470	62.2546	0.8861	0.0120	0.0228	*
New twigs endosphere 4th vs Rhizosphere 4th	1	0.0400	59.6870	0.8818	0.0110	0.0228	*
New twigs endosphere 4th vs Fruit endosphere 5th	1	0.0030	3.2304	0.2876	0.0060	0.0228	*
New twigs endosphere 4th vs Fruit episphere 5th	1	0.0032	1.7661	0.1808	0.1550	0.1710	
New twigs endosphere 4th vs Leaf episphere 5th	1	0.0226	40.5466	0.8352	0.0100	0.0228	*
New twigs endosphere 4th vs New twigs endosphere 5th	1	0.0068	8.2747	0.5084	0.0090	0.0228	*
New twigs endosphere 4th vs Rhizosphere 5th	1	0.0396	55.3632	0.8737	0.0080	0.0228	*
Rhizosphere 4th vs Fruit endosphere 5th	1	0.0538	113.6308	0.9342	0.0090	0.0228	*
Rhizosphere 4th vs Fruit episphere 5th	1	0.0367	26.3337	0.7670	0.0120	0.0228	*
Rhizosphere 4th vs Leaf episphere 5th	1	0.0577	492.3115	0.9840	0.0110	0.0228	*
Rhizosphere 4th vs New twigs endosphere 5th	1	0.0336	87.2601	0.9160	0.0100	0.0228	*
Rhizosphere 4th vs Rhizosphere 5th	1	0.0077	28.1734	0.7788	0.0060	0.0228	*
Fruit endosphere 5th vs Fruit episphere 5th	1	0.0071	4.3103	0.3501	0.0150	0.0240	*
Fruit endosphere 5th vs Leaf episphere 5th	1	0.0341	94.4345	0.9219	0.0090	0.0228	*
Fruit endosphere 5th vs New twigs endosphere 5th	1	0.0110	17.5013	0.6863	0.0070	0.0228	*
Fruit endosphere 5th vs Rhizosphere 5th	1	0.0565	108.9506	0.9316	0.0110	0.0228	*
Fruit episphere 5th vs Leaf episphere 5th	1	0.0187	14.5709	0.6456	0.0080	0.0228	*
Fruit episphere 5th vs New twigs endosphere 5th	1	0.0089	5.7496	0.4182	0.0140	0.0237	*
Fruit episphere 5th vs Rhizosphere 5th	1	0.0297	20.6525	0.7208	0.0080	0.0228	*
Leaf episphere 5th vs New twigs endosphere 5th	1	0.0271	99.2064	0.9254	0.0090	0.0228	*
Leaf episphere 5th vs Rhizosphere 5th	1	0.0343	211.4804	0.9636	0.0040	0.0228	*
New twigs endosphere 5th vs Rhizosphere 5th	1	0.0411	95.4989	0.9227	0.0060	0.0228	*

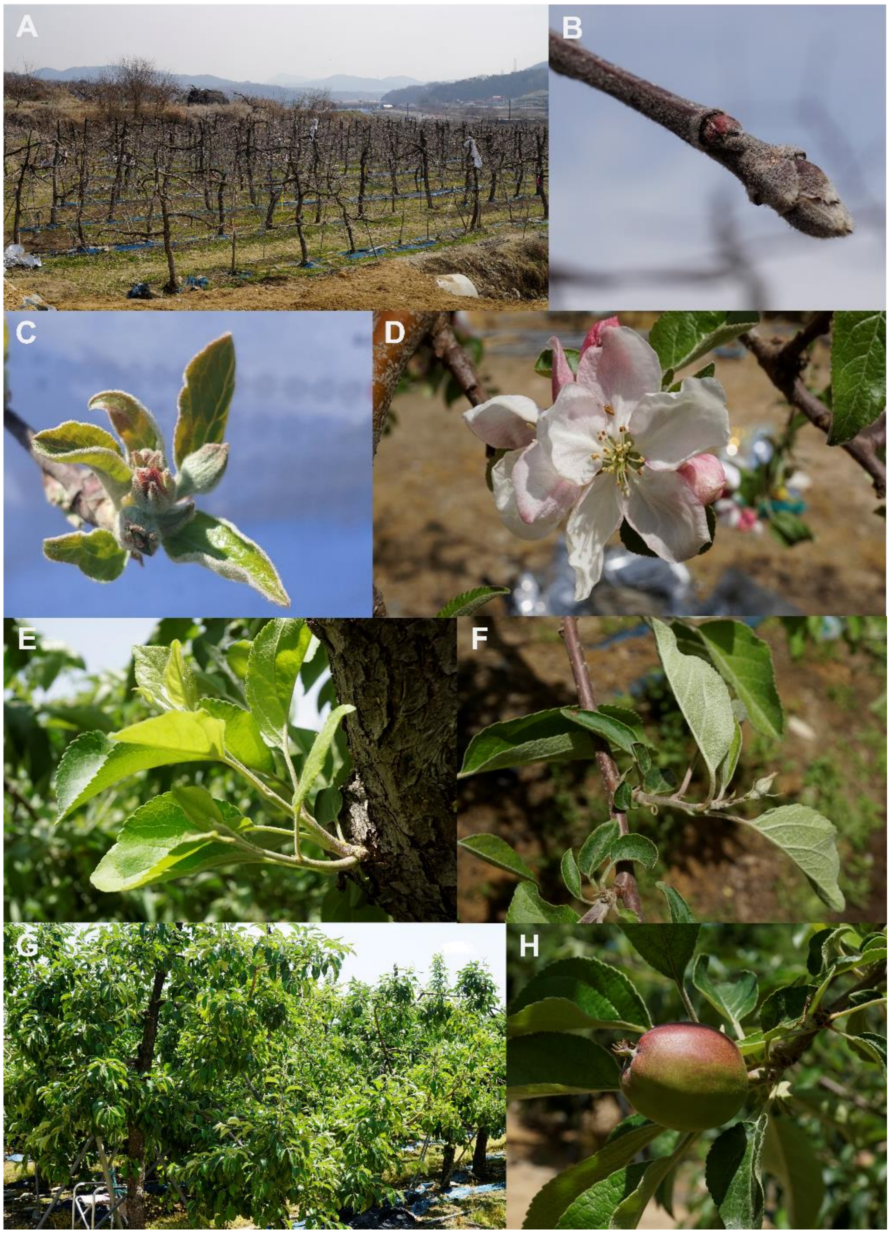

Fig. S1. Panorama and organization of sampling orchard. **A,** Schematic diagram **B,** Leaf bud organization. **C,** Flower bud organization. **D,** Flower organization. **E, F,** Twig organization of sampling orchard. **G, H,** fruit stage in the sampling orchard. Each tree is used for sampling (n = 5).

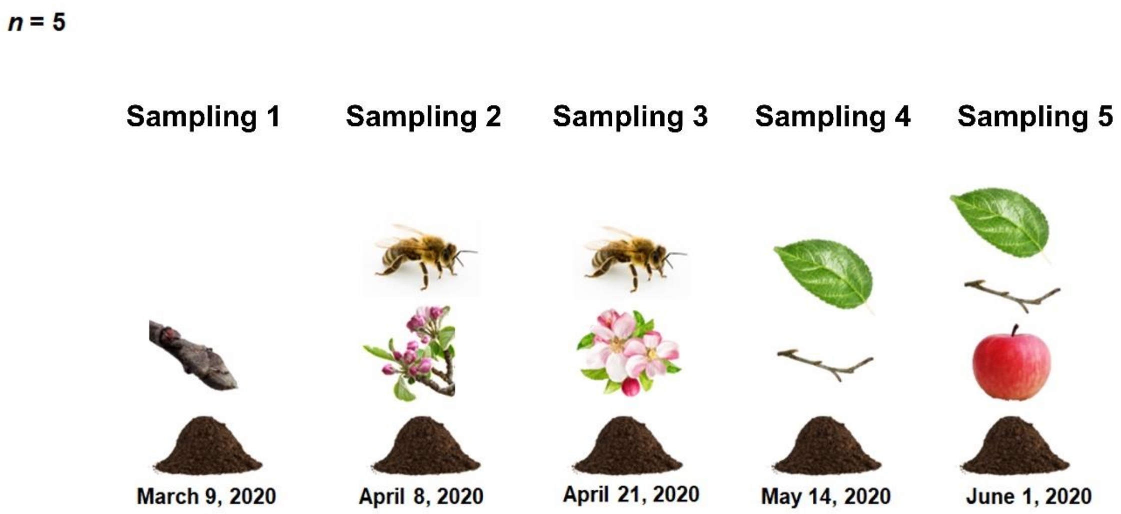

Fig. S2. Apple's seasonal development stages and sampling in this study.

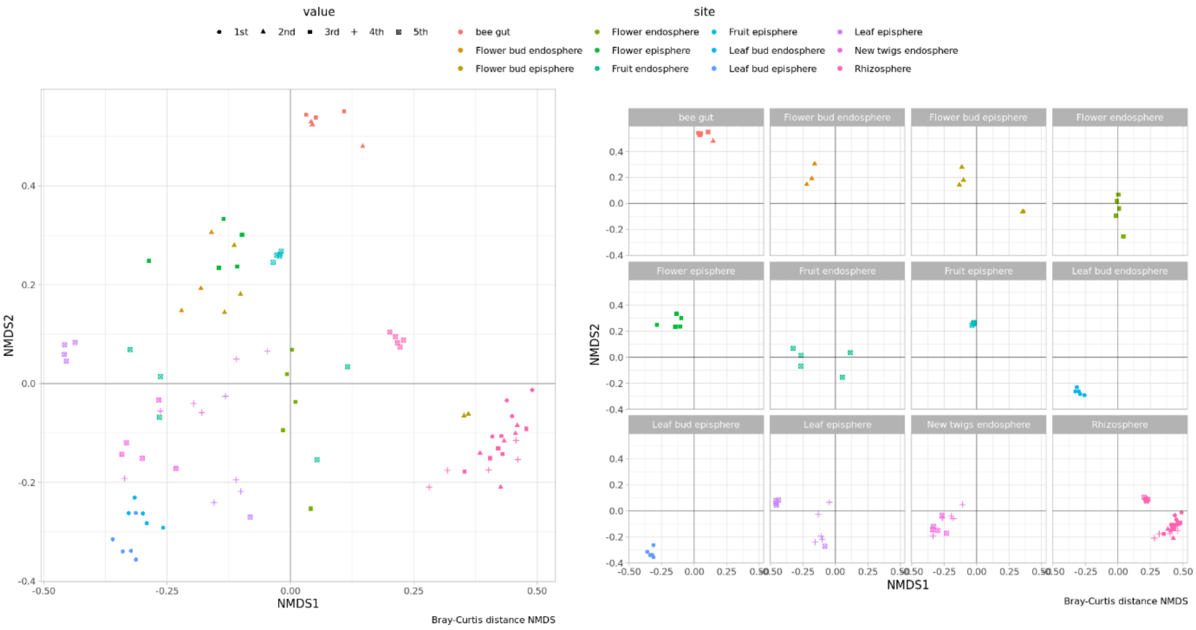

Fig. S3. Bray-Curtis distance based NMDS. The left panel displays all dots meaning each sample. The right panel, segmented by sampling site, is drawn for ease of viewing the left panel.
